# Palliative Chemotherapy: Does It Only Provide False Hope? The Role of Palliative Care in a Young Patient With Newly Diagnosed Metastatic Adenocarcinoma

**Published:** 2017-05-01

**Authors:** Lisa Doverspike, Sharyn Kurtz, Kathy Selvaggi

**Affiliations:** 1 Butler Health System, Palliative Care Division, Butler, Pennsylvania;; 2 Dana-Farber Cancer Institute, Department of Medical Oncology, Boston, Massachusetts;; 3 Butler Health System, Palliative Care Division, Butler, Pennsylvania

## Abstract

**Case Study**

A 48-year-old female with recent diagnosis of adenocarcinoma of unknown origin and metastatic disease to the peritoneum initially presented to a nearby academic hospital with abdominal pain. She underwent exploratory laparotomy with tumor debulking surgery at that time. Shortly thereafter, she was readmitted to the same hospital with evidence of partial small bowel obstruction and treated conservatively with bowel rest, nasogastric (NG) tube placement, and intravenous (IV) fluid administration. Eventually the NG tube was removed, and she was discharged home. The following day, she received cycle one of palliative chemotherapy with cisplatin and gemcitabine at her local outpatient oncology clinic. She experienced persistent nausea and intermittent vomiting throughout the night and presented to our local community hospital for evaluation.

At the time of admission, she was passing minimal flatus and had passed only a small bowel movement that morning. She had experienced nausea, vomiting, and poor oral intake for over a week. Other presenting symptoms included mild to moderate abdominal pain involving the upper abdomen. Upon evaluation, abdominal x-ray revealed dilated loops of small bowel, consistent with partial small bowel obstruction. An NG tube was placed, and the patient’s symptoms were initially improved with bowel rest.

Her medical history was significant for pulmonary embolism detected at the time of her adenocarcinoma diagnosis, and she was on oral anticoagulation and home oxygen. She also had a history of depression and total abdominal hysterectomy/bilateral salpingo-oophorectomy (TAH/BSO) due to fibroids. Her social history revealed she was an office worker and married with two sons, ages 18 and 24. The 18-year-old son lived at home with the patient and her husband. The patient was eagerly awaiting the birth of a granddaughter, due in a few weeks’ time. Her mother and father were also present daily during her hospitalization and were a major source of support for her and her family.

At the time of hospital admission, a surgical team consultation concluded she was not a candidate for palliative surgery due to extensive disease burden. She was seen in consultation by medical oncology, who recommended resuming chemotherapy once the acute partial small- bowel obstruction resolved.

**Palliative Care Consult**

A palliative care consultation was requested to assist with symptom management, including pain and nausea relief. At the time of consultation, the patient appeared in mild distress due to abdominal pain and distention. Vital signs were stable. Physical exam was significant for absent bowel sounds and a mildly distended but nontender abdomen. The NG tube was in place, draining bilious gastric fluid. She had mild nonpitting edema involving the bilateral lower extremities. Discussion with the patient revealed values consistent with improving symptoms and extending life expectancy as long as possible. The patient expressed wishes for "aggressive treatment," which she defined as continuation of chemotherapy and full resuscitation.

The palliative care team discussed symptom management options with the patient. Nonsurgical management of partial bowel obstruction was continued, including bowel rest, NG tube decompression, and IV fluids. Pain was controlled initially with IV morphine as needed. After symptom improvement and evidence of bowel function recovery, the NG tube was removed. However, after a short time, she required NG tube replacement due to recurrent nausea and vomiting. Discussion was initiated with the patient, who opted for placement of a venting gastrostomy tube (G-tube) and total parenteral nutrition (TPN), with the goal of symptom relief and administration of nutrition, which would allow for continuation of chemotherapy.

During placement of the venting G-tube, the gastroenterology (GI) team noted extensive tumor involving the stomach, which made placement of the tube difficult. Additionally, anticoagulation was held during G-tube placement, and postoperatively, the patient experienced acute, right-sided chest pain and shortness of breath. Computed tomography (CT) scan with pulmonary embolus (PE) protocol revealed a new PE, and anticoagulation was changed to enoxaparin. Shortly thereafter, she became febrile and developed leukocytosis. Blood cultures revealed coagulase-negative staphylococcus from a Port-a-Cath source. She was treated with appropriate antibiotic therapy; however, follow-up blood cultures revealed persistent coagulase-negative staphylococcus bacteremia. Her indwelling Port-a-Cath was removed. After appropriate antibiotic therapy, a peripherally inserted central catheter line was inserted and TPN restarted.

**Reinstituting Palliative Chemotherapy**

Palliative care discussion with the patient confirmed her desire to reinstitute palliative chemotherapy, with the goal of restoring bowel function and returning home. Chemotherapy was resumed on day 15, despite concerns and even objections from several nursing staff members. The patient experienced treatment side effects, including prolonged thrombocytopenia. A platelet function antibody returned positive, consistent with heparin-induced thrombocytopenia. Enoxaparin was discontinued, and fondaparinux (Arixtra) was initiated. Platelet count recovered shortly thereafter.

The patient required intense symptom management due to intractable abdominal pain and nausea and vomiting despite adequate venting G-tube decompression. Medical management was maximized with antiemetics, antisecretory agents, steroids, and antipsychotic agents, and symptoms eventually improved after cycle 2 of chemotherapy. Thereafter, the patient was discharged home. At the time of discharge, her symptoms were well controlled on minimal pain medications. She was still experiencing intermittent nausea but was passing flatus.

By reducing the tumor burden, chemotherapy significantly improved her quality of life. She spent a total of 7 weeks in the hospital. During that time, she received two cycles of chemotherapy plus best supportive care and symptom management. Despite intermittent nausea and vomiting, administration of palliative chemotherapy allowed this patient to achieve her primary goals, which included returning home to her family and regaining some bowel function. Over the next several months, she received several more cycles of outpatient palliative chemotherapy. She experienced mild to moderate nausea and intermittent vomiting despite G-tube venting. Eventually, her disease progressed, and the patient chose to forgo any further intervention or chemotherapy. She was enrolled in hospice care and died comfortably at home surrounded by her family.

Malignant bowel obstruction is a common and challenging issue that affects 20%–50% of patients with metastatic ovarian cancer and 10%–28% of patients with metastatic colorectal cancer at some point in their disease course (Ripamonti, Easson, & Gerdes, 2008). Patients present at varying stages in their disease course, and therefore, treatment courses and outcomes vary. Although surgical intervention may be the preferred treatment in selected patients, many are deemed not to be surgical candidates due to the extent of disease, peritoneal carcinomatosis, or poor performance status (Ripamonti et al., 2008). Interventions such as gastrointestinal tract stenting or venting gastrostomy tube placement may also be helpful. Medical management includes gastric decompression with a nasogastric (NG) tube, fluid and electrolyte repletion, and nausea management with antiemetics and antisecretory agents.

## PALLIATIVE STRATEGIES

Palliative strategies for minimizing pain and nausea in malignant bowel obstruction include the use of intravenous (IV) opioids, sublingual or injectable forms of antiemetics, antipsychotics, and antisecretory medications. Agents such as lorazepam and haloperidol may be useful to relieve nausea. Somatostatic analogs such as octreotide (Sandostatin) function by inhibiting the secretion of gastric and small bowel fluid and pancreatic enzymes. These medications also inhibit neurotransmission in peripheral nerves of the gastrointestinal tract, leading to decreased peristalsis and decreased splanchnic blood flow (Von Gunten & Muir, 2015). These therapies may be effective for symptomatic relief of pain, nausea, and the burdens of high NG tube output. Corticosteroids are commonly used to relieve nausea in a number of settings by way of both central and peripheral effects. Dexamethasone is used to decrease gut inflammation and reduce bowel wall edema, which is often caused by an obstruction. Despite its prokinetic effects, it is recommended to avoid the use of the antiemetic, metoclopromide, in cases of complete bowel obstruction, as this may exacerbate crampy abdominal pain (Von Gunten & Muir, 2015).

To maximize symptomatic relief, it is often necessary to combine the benefits and mechanisms of action of several medications. In this patient’s case, her nausea was refractory to ondansetron, lorazepam, prochlorperazine, and even dexamethasone. She did find some relief from octreotide injections and continued to derive benefit from gastrostomy tube (G-tube) venting. All of these medical interventions were used in conjunction with palliative chemotherapy. However, the best predictor of improved patient outcome is tumor sensitivity to platinum-based chemotherapeutic agents (Bryan, Radbod, & Berek, 2006).

Palliative chemotherapy is intended not to cure, but instead to improve or control symptoms, as well as extend life expectancy. It is important to understand the response rate and expected or median duration of response while recognizing the potential treatment burden (Weissman, 2015).

In the case described previously, the patient was chemotherapy naive. The primary site of her tumor was unknown. Review of pathology revealed adenocarcinoma with immunohistochemical stains most consistent with an upper gastrointestinal origin. Based on this information, the regimen of cisplatin and gemcitabine was carefully selected for this patient as a commonly active regimen in upper gastrointestinal malignancies. We believed her tumor would respond to chemotherapy, and decrease in tumor burden would be essential in reducing her symptoms. Additionally, her young age and lack of comorbidities contributed to her candidacy for chemotherapy.

Understanding the patient’s treatment goals and the projected response rate to de novo chemotherapy is essential in appreciating the rationale for treatment. Treatment of her underlying malignancy was the primary means of controlling and improving symptoms related to malignant bowel obstruction. Incorporating care from palliative care specialists to address the multitude of symptoms that are commonly associated with treatment of advanced cancer is paramount.

## MORAL DISTRESS

The complexities of this case go far beyond the medical details, treatment complications, and care decisions described previously. This case also highlights an example of moral distress, in which the nursing staff felt morally at odds with the treatment recommendations provided by the palliative care and oncology teams. The nursing staff voiced their discomfort regarding administering chemotherapy to a patient with a terminal prognosis. This patient’s young age and relatability also contributed to challenges for all those who participated in her care. Concerns regarding the care of this patient became exceedingly apparent as her length of stay increased and her medical care became more complicated. At times, her condition appeared to decline irreversibly. It was during these episodes that the nursing staff asked, "Why are we administering chemotherapy?" Statements such as "Chemotherapy makes people feel worse" and "This patient needs hospice" communicated the staff’s discomfort with the treatment she was receiving. Staff also had concerns that the patient did not understand her diagnosis and that she had false hope regarding the benefits of chemotherapy. There were also staff misconceptions about the role of palliative care. One nurse commented, "Isn’t palliative care just for end-of-life transition?"

When caring for medically complex patients, nurses may be asked by interdisciplinary team members to perform deeds that feel morally compromising. Moral distress may be experienced by any member within the interdisciplinary care team, although moral distress is particularly common among bedside hospital staff and those involved in end-of-life patient care (Crippen, 2016; Whitehead, Herbertson, Hamric, Epstein, & Fisher, 2015).

Situations of moral distress consist of either internal (e.g., personal) or external (e.g., institutional) constraints that prevent a clinician from acting in a manner that is consistent with his/her moral values (Epstein & Hamric, 2009). In other words, the clinician knows the right thing to do but feels unable to do it.

This case highlights the importance of communication among interdisciplinary team members. Palliative care education, interdisciplinary team meetings, and additional nurse support helped alleviate the ethical concerns raised by this patient’s nurse caregivers. Debriefing sessions were informally held during this patient’s admission as well as after her discharge to facilitate open discussion and continued education.

## CONCLUSION

Palliative care clinicians can have a pivotal role in alleviating angst in emotionally and medically challenging situations. The role of the palliative care team in this case was multifactorial: We elucidated the patient’s wishes and care goals, advocated for the continuation of palliative chemotherapy and aggressive interventions to control symptoms, and facilitated discussions with health-care providers to help alleviate their moral distress.
